# Relationships Between *Candida auris* and the Rest of the *Candida* World—Analysis of Dual-Species Biofilms and Infections

**DOI:** 10.3390/pathogens14010040

**Published:** 2025-01-08

**Authors:** Monika Janeczko, Tomasz Skrzypek

**Affiliations:** 1 Department of Molecular Biology, Faculty of Medicine, The John Paul II Catholic University of Lublin, Konstantynów 1i, 20-708 Lublin, Poland; 2 Department of Biomedicine and Environmental Research, Faculty of Medicine, The John Paul II Catholic University of Lublin, Konstantynów 1j, 20-708 Lublin, Poland; tomasz.skrzypek@kul.pl

**Keywords:** dual-species biofilm, candidiasis, *Galleria mellonela*, hyphae, mixed infection, SEM

## Abstract

In this study, we investigated the interactions between *Candida auris* and *C. albicans*, *C. tropicalis*, *C. glabrata*, and *C. krusei* in mixed infections. Initially, these interactions were studied qualitatively and quantitatively in dual-species biofilms formed in vitro. The MTT assays, determination of the total CFU/mL, and SEM analysis showed that *C. auris* interacted differentially with the other *Candida* spp. during the dual-species biofilm formation. Depending on the stage of the biofilm development, *C. auris* was found to be a particularly dominant species during its interaction with the *C. krusei* biofilms but significantly submissive in the *C. auris-C. albicans* biofilms. These studies were then extended to in vivo host models of experimental candidiasis. *G. mellonella* larvae were inoculated with monotypic and heterotypic suspensions of *Candida*. The survival rates and quantification of fungal cells in the hemolymph showed that the highest mortality was exhibited by larvae in the *C. auris-C. albicans* co-infection (100% mortality after 36 h). The CFU/mL values of *C. auris* from the larval hemolymph were lower in the interactive groups compared to the mono-species group. As a newly emerging species, *C. auris* persists in environments in the presence of other *Candida* species and is involved in both competitive and noncompetitive interactions with other *Candida* species during biofilm formation and development of experimental candidiasis.

## 1. Introduction

Fungal infections pose a worldwide threat to health and life, especially in immunocompromised patients. Due to their distribution, extent, and course, fungal infections are divided into two types: (a) superficial infections of the skin and mucous membranes and (b) invasive fungal infections attacking the bloodstream, lungs, liver, kidneys, and central nervous system [1]. The occurrence of invasive fungal infections is particularly dangerous in patients with comorbidities, in subjects with reduced immunity, after organ transplantation, those undergoing chemotherapy, and in patients with HIV/AIDS or autoimmune diseases. A consequence of fungal infections is the disturbing death toll of 1.7 million annually worldwide [2,3]. *Candida* species are the causative agent of most fungal infections in humans. *C. albicans*, *C. glabrata*, *C. parapsilosis*, *C. tropicalis*, *C. krusei*, and *C. auris* are pathogens responsible for 95% of infections. Invasive infections caused by *Candida* species are mainly associated with the healthcare environment and are the fourth most common cause of nosocomial infections [4].

*C. auris* was first isolated from the ear canal of a 70-year-old Japanese woman at the Metropolian Geriatric Hospital in Tokyo in 2009. By 2021, *C. auris* infections have been reported in 44 countries on six continents around the world [5]. *C. auris* infections mainly affect hospitalized patients. Transmission in the hospital environment probably occurs through contact with contaminated surfaces or equipment and may be transmitted from person to person through contact with contaminated skin. It has been proven that *C. auris* has the ability to survive on biotic and abiotic surfaces for several weeks and even months [6,7,8]. The risk factors associated with *C. auris* infections are similar to those for other *Candida* species. The most important are old age, diabetes, surgery, catheterization of the main veins, immunocompromised state, parenteral nutrition, hemodialysis, neutropenia, chronic kidney diseases, and the use of broad-spectrum antibiotics or antifungal drugs [8,9,10]. Interestingly, it has also been proven that exposure to *C. auris* infections is associated with the occurrence of diarrhea and treatment with broad-spectrum antibiotics—tetracyclines, e.g., second-generation tetracyclines, including minocycline and tigecycline [11].

*C. auris* infections have a virtually identical clinical picture as other infections with other *Candida* species. The infections mainly affect the blood and are systemic (fungemia) [12]. Unlike infections with other *Candida* species, which are usually endogenous through autoinfections from the host microflora, *C. auris* is a source of mainly exogenous infections. *C. auris* is very often transmitted to patients from contaminated environments, equipment, and tools. Laboratory tests have confirmed its survival on infected surfaces from seven days to four weeks [13]. In a study monitoring the survival of planktonic cells or cells in the form of biofilm on glass, plastics, fabrics, steel, and wood, despite daily disinfection, it was found that *C. auris* is able to stay alive for up to 3 weeks. This fungus was isolated from the external ear, nasal cavity, vulva and vagina, urinary tract, cerebrospinal fluid, surgical and burn wounds, skin abscesses related to catheter insertion, respiratory tracts, and infected bone samples [8,9]. Moreover, it has been evidenced that colonization of anatomical sites is possible in infected patients, including the groin, armpits, rectum, nose, mouth, and throat. Body colonization is also known to persist for 1–3 months after initial infection [14]. According to the Centers for Disease Control and Prevention (CDC), the mortality rate associated with invasive fungal infections is 30%. *C. auris* infections have a very high mortality rate, although they are not more common than *C. albicans* infections. For example, the rates reported in Venezuela, India, and Panama were 28%, 50%, and 78%, respectively [6,8].

*C. auris* currently represents the greatest clinical challenge in the control of nosocomial infections worldwide. The high mortality and frequency of invasive infections are related to the virulence factors of this pathogenic yeast. Although the causes of the pathogenicity of *Candida* species are very well understood, they are still under intensive research in the case of *C. auris*. Many virulence features of *C. auris* resemble those of *C. albicans* and include the ability to form pseudohyphae and biofilms, adherence to biotic and abiotic surfaces, cellular aggregation, secretion of proteinases, phospholipases, hemolysins, uptake of nutrients and iron, and the ability to rearrange the composition of the cell wall [8,15].

Among the virulence factors distinguishing *Candida* species are the formation of hyphae and growth in the form of biofilms. Hyphae represent an important phase in biofilm formation and in the disease process and can cause tissue damage by invading mucosal epithelial cells, then leading to invasive infection. The morphology of *C. albicans* (yeast or hyphae) is influenced by several environmental stimuli, including temperature, pH, nutrient availability, or quorum sensing mechanisms. The importance of hyphal growth is demonstrated by the fact that non-filamentous strains of *C. albicans* are avirulent [1,4]. Hyphae secrete the peptide toxin candidalysin, which is crucial for the destruction of host cell membranes. The peptide is derived from a precursor protein encoded by the gene *ECE1*, which is strongly induced during hyphal growth [16]. Candidalysin causes necrotic damage to infected human epithelial cells, resulting in subsequent translocation across intestinal barriers. The toxin also provokes an immune response, including activation of the NLRP3 inflammasome, recruitment of neutrophils, and release of proinflammatory cytokines. In infected oral epithelial cells, candidalysin initiates phosphorylation of the epidermal growth factor receptor (EGFR) and activation of the Eph2-EGFR signaling pathway. In addition, candidalysin induces the release of alarmin and antimicrobial peptides in epithelial cells [17]. *C. albicans* also produce other proteins associated with hyphal properties including cell wall-dissolving enzymes and adhesion factors, such as secreted aspartic proteases (Saps) and agglutinin-like sequences (Als) that allow *C. albicans* to adhere to, penetrate, and damage epithelial cells [1,4]. Biofilms are multicellular structures surrounded by a layer of mannan-glucan polysaccharides, which constitutes a barrier to the external environment. The formation of *Candida* biofilms has been observed on many surfaces, including living surfaces (mucous membranes, organs, blood vessels) and inanimate surfaces, often in the case of medical devices in contact with the patient’s body (stents, valves, implants, endotracheal tubes, pacemakers, and many types of catheters) [18]. *Candida* can form biofilm within 38–72 h. Biofilm growth is a complex process consisting of several stages, the first of which is the adhesion of yeast to the surface. The next stage of biofilm development is cell proliferation and the early stage of the formation of hyphae. As a result, the biofilm surrounded by a polysaccharide matrix matures. The final stage of biofilm development is dispersion, during which some yeast cells escape from the biofilm into the environment [19]. Yeast proliferation and biofilm formation are also influenced by their response to homeostasis and variability of the host (changes in mucosal pH or changes in nutrition) and the state of the host immune system. Biofilms constitute a difficult obstacle for the immune system cells of the host and antifungal drugs. Additionally, cells in biofilms are distinguished by the increased activity of molecular pumps that effectively remove antifungal substances (efflux pumps) [20]. Biofilm formation varies depending on the *Candida* species. Compared to *C. albicans*, *C. auris* grows less frequently in the form of biofilms, and the structures themselves are much thinner. Transcriptomic analyses have shown that the expression of adhesion proteins, including CSA1, IFF4, PGA26, and PGA52, increases in the first stage, i.e., adhesion of *C. auris* cells. Moreover, Als proteins play an important role in cell adhesion. Interestingly, *C. auris* has significantly fewer ALS and other adhesin genes than *C. auris*, which may explain why *C. auris* biofilms are less common and weaker [21]. However, some strains of *C. auris* may have a greater tendency to produce biofilm, which mainly applies to representatives of clade III (located in South America). The strains belonging to this clade show higher expression of adhesins and are more likely to form aggregates, leading to the formation of biofilm [22]. Moreover, during biofilm maturation, an increase in the expression of ABC (ATP-Binding Cassette Superfamily) transport proteins, such as CDR1, SNQ2, and YHD3, and MFS (Major Facilitator Superfamily) proteins, mainly MDR1 and RDC3, is observed [23,24]. ABC and MFS proteins actively transport endogenous and exogenous substrates across biological membranes; therefore, they play an important role in regulating many physiological functions necessary for body homeostasis and the response to drugs. Hence, increased efflux of antifungal drugs is observed in *C. auris* cells forming biofilms, and mature biofilms show resistance to all commonly used antifungal drugs [24]. Importantly, biofilms have been evidenced to be a factor contributing to the spread of *C. auris* infections in hospitals [25].

It is known that *Candida* species, especially *C. albicans*, form polymicrobial biofilms, interacting with bacteria, e.g., *Staphylococcus aureus*, *Streptococcus mutans*, *Streptococcus gordonii*, *Staphylococcus epidermidis*, *Acinetobacter baumannii*, Bacteroides fragilis, Bacteroides vulgatus, Clostridium perfringens, *Pseudomonas aeruginosa*, and *Lactobacillus* spp. [26,27,28,29,30,31,32,33,34,35]. Interactions between *C. albicans* and microorganisms in biotic and abiotic media are diverse and complex, including synergistic, antagonistic, and neutral relationships [36]. *Candida*-*Candida* co-existence within biofilms has also been demonstrated [37,38,39,40,41,42]. *C. auris*, as an emerging species, is subject to intensive research in terms of virulence characteristics, including the ability to form a biofilm and interspecies interactions. It is unclear what the relationship is between *C. auris* and other common *Candida* species coexisting in the environment and human host. Despite its close phylogenetic relationship to other pathogenic *Candida* species, *C. auris* has many unique features in its biology, genetics, epidemiology, drug resistance, virulence, and ability to adapt to various biotic and abiotic environments, which may shape these interspecies interactions [8,13,14]. This study aimed to assess the formation of both single- and dual-species biofilms consisting of *C. auris* and four *Candida* species, *C. albicans*, *C. glabrata*, *C. krusei*, and *C. tropicalis,* and the relationships between these species in mixed biofilms. Moreover, in this report, we evaluated the interactions between *C. auris* with other *Candida* species in mixed infections using *G. mellonella* as a host model.

This study was designed to determine the ability of *C. auris* to persist in the environment in the presence of other clinically important *Candida* species in dual-species biofilms and infections. To our knowledge, this is the first study to describe the interspecies relationships of *C. auris* with *C. albicans*, *C. tropicalis*, *C. glabrata*, or *C. krusei* in vitro and in vivo models.

## 2. Materials and Methods

### 2.1. Candida Species, Culture Media, and Chemicals

Reference strains of *Candida auris* (ATCC MYA-5001), *Candida albicans* (ATCC 10231), *Candida glabrata* (ATCC 15126), *Candida krusei* (ATCC 14243), and *Candida tropicalis* (ATCC 13803) were obtained from the American Type Culture Collection (ATCC, Gaithersburg, MD, USA). The fungal strain cultures were grown in YPD medium (1% yeast extract, 2% peptone, 2% dextrose), pH 5.6, and RPMI 1640 medium supplemented with L-glutamine and buffered with morpholinepropanesulfonic acid (MOPS) (Sigma-Aldrich, St. Louis, MO, USA) at 0.165 M, pH 7.0, and CHROMagar Candida Plus medium (Biomaxima, Lublin, Poland). Fetal bovine serum (FBS), 3-[4,5-dimethylthiazol-2-yl]-2,5 diphenyl tetrazolium bromide (MTT), menadione, phosphate-buffered saline (PBS), poly-L-lysine (PLL), and other chemicals were purchased from Sigma-Aldrich (Sigma-Aldrich, St. Louis, MO, USA).

### 2.2. Biofilm Formation and Quantification

The *Candida* overnight cultures grown in YPD broth were diluted to 0.5 McFarland standard in RPMI 1640 medium supplemented with 10% FBS. In total, 100 mL (individually) or 50 mL (for each strain, in the case of dual-species biofilm) of the *Candida* suspension was added to the wells of polystyrene 96-well microtitre plates (Corning Inc., Corning, NY, USA). After incubation, the medium in the wells was removed and the plates were washed three times with sterile PBS. For the assessment of the metabolic activity of adherent *Candida* cells, 200 µL of the MTT solution (40 µL of 1 mg/mL MTT, 2 µL of 0.4 mM menadione, and 158 µL of PBS) was added to each well of the microtitre plates. After incubation at 37 °C for 3 h, absorbance at 570 nm was measured using a microplate reader (BioTek Synergy H1, Biokom, Janki, Poland). Biofilm formation was quantified at 6 h, 12 h, 18 h, 24 h, 36 h, and 48 h. *C. auris* was used as the control strain in all the experiments. In addition, aliquots (100 mL) of scrapped biofilm were sonicated, serially diluted in PBS, and plated on CHROMagar Plus Candida medium. The plates were incubated at 37 °C for 48 h. After this time, colony-forming units (CFUs) of each *Candida* species in the mono-species or dual-species biofilms were determined. The CFU values of the *Candida* species that formed the biofilm (mono- and dual-species) were statistically compared with the CFU of the mono-species *C. auris* biofilm produced at each time point (6 h, 24 h, and 48 h).

### 2.3. Scanning Electron Microscopy (SEM)

Mono-species or dual-species biofilms were formed on poly-L-lysine (PLL)-coated glass. Clean glass coverslips were immersed in a 0.1% PLL solution for 10 min and air-dried overnight, rinsed with sterile deionized water, and air-dried. The cultures were grown overnight in YPD medium and then diluted to an optical density of 0.5 McFarland standard in RPMI 1640 with 10% FBS. After incubation at 37 °C for 12 h and 24 h, the glass coverslips were removed from the Petri dishes, and the dishes were rinsed with sterile water. Then, the samples were rinsed for 2–3 min with a saline solution and fixed in 4% (*v*/*v*) buffered formalin for 1 h. After fixation, the probes were washed in water and dehydrated in a series of alcohol: 30%, 50%, 70%, 90%, and finally in absolute ethanol. The samples were dried in a critical point dryer (CPD, Polaron Range, CPD 7501, ELO SERWIS, Warsaw, Poland)) and sputter coated (Polaron Range, Sputter Coater 7620, ELO SERWIS, Warsaw, Poland) with a 20 nm layer of gold. The biofilms were observed using a LEO 1430VP scanning electron microscope (Zeiss, ELO SERWIS, Warsaw, Poland) at an accelerating voltage of 15 kV, with an average working distance of 8–9 mm.

### 2.4. Candida Interactions in the Galleria mellonella Model

This study was conducted according to the methodology described by Mylonakis et al. (2005) and Cowen et al. (2009) with some modifications [43,44]. Sixteen randomly selected *G. mellonella* larvae of similar weight and size (250–300 mg) were used in each study group. Two control groups were included in the tests: one group was inoculated with PBS to allow observations of larval death due to physical trauma and the other group did not receive the injection as a control for general viability. *Candida* cell suspensions were prepared from cultures in liquid YPD medium at 37 °C for 20 h. The cells were then centrifuged at 2000× *g* for 10 min, and the supernatant was discarded. The cell pellet was dissolved in PBS and homogenized in a tube shaker for 30 s, repeating this procedure twice. Cell densities were determined using a densitometer. In the case of mixed infections, larvae were infected with 1 × 10^6^ CFU/larva of *C. albicans* and 1 × 10^6^ CFU/larva of the non-albicans species (*C. glabrata* or *C. krusei*). In the case of single infections, larvae were infected with 2 × 10^6^ CFU/larva. *Candida* suspensions were injected into the hemolymph of each larva via the last two prolegs. After inoculation, the larvae were stored in plastic containers at 37 °C, and the number of killed *G. mellonella* was recorded every 12 h for 96 h of the experiment. Larvae were considered dead when they did not respond to touch.

### 2.5. Candida Species Quantification in G. mellonella Hemolymph

To determine the numbers of each *Candida* species in the *G. mellonella* infections, the larvae were infected with 1 × 10^6^ CFU/larva of *C. auris* and 1 × 10^6^ CFU/larva of non-auris species in different prolegs (mixed infections). The larvae also received two injections: one with 1 × 10^6^ CFU/larva of *Candida* and the other with PBS (single infections). Fungal cells were extracted from the hemolymph of *G. mellonella* immediately after inoculation (0) and at intervals of 6, 12, and 18 h after infection of the larvae with the different *Candida* species. Five larvae were used to collect sufficient amounts of hemolymph. The surviving larvae were squeezed to collect hemolymph (approximately 100 μL). The extracted hemolymph was then serially diluted in sterile PBS. From each dilution, 100 μL was transferred to CHROMagar Candida Plus medium plates. The plates were incubated for 48 h at 37 °C, and then colonies were counted and CFU/mL values were determined. The experiment was performed in triplicate.

### 2.6. Statistical Analysis

All data are expressed as a mean ± SD (standard deviation) of three duplicates from two independent experiments. To assess normality, the Shapiro–Wilk test was used. Statistical significance between the treated and control groups was analyzed by Student’s *t*-test using GraphPad Software version 9.1.1. (San Diego, CA, USA). In the MTT assay for the determination of the metabolic activity of dual-species biofilms at each time point of biofilm maturation, the optical density of the mixed biofilms was compared with the optical density of *C. auris* or non-auris *Candida* species. In all the other tests, at each time point during the experiments, the test samples were compared with the reference samples from the mono-species culture or *C. auris* monospecies infection. A *p* value < 0.05 was considered statistically significant.

## 3. Results

### 3.1. Analysis of Mono-Species Biofilms In Vitro

**Figure 1:**
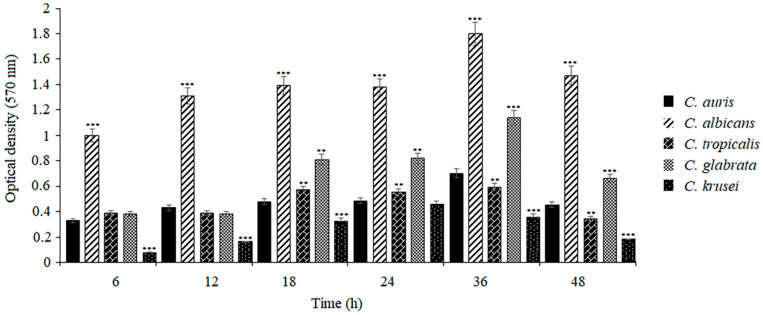
Biofilm formation by *Candida* mono-species on microtitre plates. Biofilm viability was quantified by the MTT assay. At each time point of biofilm maturation, the optical density of *Candida* mono-species biofilms was compared with the optical density of *C. auris* (black columns, ** *p* < 0.01; *** *p* < 0.001) by a Student’s *t*-test. Data are the means ± SD of experiments performed in three duplicates and repeated two times.

The intensity of biofilm growth of individual *Candida* species was determined using the MTT method. In this study, the metabolic activity of yeast cells was measured at time points after 6, 12, 18, 24, 36, and 48 h of growth. As shown in Figure 1, *C. auris* showed a significantly weaker ability to form biofilm than *C. albicans* (*** *p* < 0.001). Compared to *C. tropicalis*, these differences were smaller (* *p* < 0.05, ** *p* < 0.01). In the case of *C. glabrata*, the biofilm metabolic activity increased significantly within 18–48 h of incubation, compared to the metabolic activity of the *C. auris* biofilm (** *p* < 0.01, *** *p* < 0.001). In turn, the weakest biofilm growth was exhibited by *C. krusei*, which remained at the same low level for 48 h of the experiment (*** *p* < 0.001). The highest metabolic activity of the biofilm cells of all the species was shown after 36 h of culture, while a decrease in these activities was observed at 48 h of incubation.

### 3.2. Analysis of Dual-Species Biofilms

**Figure 2:**
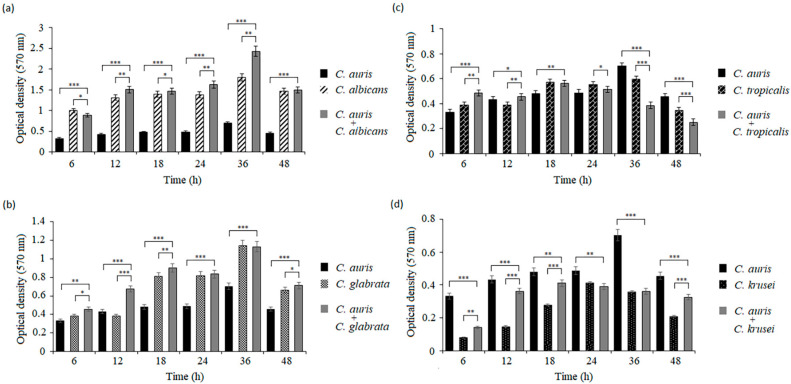
Biofilm formation of *Candida* dual-species on microtiter plates: (**a**) *C. auris* with *C. albicans*, (**b**) *C. auris* with *C. glabrata*, (**c**) *C. auris* with *C. tropicalis*, and (**d**) *C. auris* with *C. krusei*. Biofilm viability was quantified by the MTT assay. At each time point of biofilm maturation, the optical density of mixed biofilms was compared with the optical density of *C. auris* or non-auris *Candida* species (* *p* < 0.05; ** *p* < 0.01; *** *p* < 0.001) by Student’s *t*-test. Data are the means ± SD of experiments performed in three duplicates and repeated three times.

Compared with the mono-species *C. auris* biofilms, the dual-species *C. auris*/non-auris *Candida* biofilms had significantly different metabolic activities (Figure 2). In the case of the co-cultures of *C. auris* with *C. albicans* or *C. glabrata*, these biofilms were very active metabolically. The *C. auris*/*C. albicans* biofilms showed significantly higher metabolic activity already after 6 h, compared to the mono-species biofilms of *C. auris* (*** *p* < 0.001). This tendency was continued for 48 h of biofilm growth. Additionally, the dual-species biofilm activities were comparably high as the metabolic activities of the biofilms of single *C. albicans* (Figure 2a). After 48 h, a decrease in metabolic activity was observed in both mono-species and mixed biofilms. In the case of the *C. auris*/*C. glabrata* biofilms, different metabolic activities were observed within 12–48 h, compared to the *C. auris* mono-species biofilms (* *p* < 0.05, ** *p* < 0.01, *** *p* < 0.001). In turn, compared to the *C. glabrata* biofilms, these differences were insignificant at 24 h and 36 h (Figure 2b). The *C. auris*/*C. tropicalis* biofilms showed higher metabolic activity than the reference biofilms of *C. auris* within 6–18 h incubation (*** *p* < 0.001, * *p* < 0.05, ** *p* < 0.01). In contrast, these dual-species biofilms showed significantly lower metabolic activity after 36 h and 48 h, compared to the mono-species *C. auris* biofilms (*** *p* < 0.001) (Figure 2c). In turn, completely different relationships were observed in the case of the mixed *C. auris* and *C. krusei* biofilms. The dual-species biofilms showed significantly lower metabolic activities already from 6 h to 48 h of incubation, compared to the mono-species *C. auris* biofilms (*** *p* < 0.001), but their metabolic activities were significantly higher than those of the mono-species *C. krusei* biofilms (** *p* < 0.01, *** *p* < 0.001) (Figure 2d).

### 3.3. Relationships Between C. auris and Other Candida Species in Biofilms

**Figure 3:**
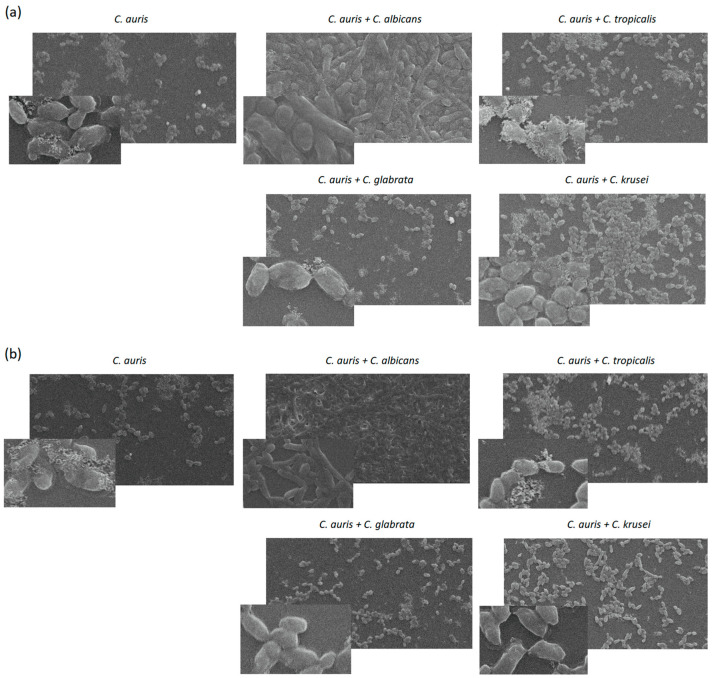
Representative scanning electron microscopy images of dual-species *Candida* biofilms compared with the mono-species *C. auris* biofilm after 24 h (**a**) and 48 h (**b**) of incubation. Magnification: 2000× (larger photo) and 5000× (smaller photo).

The SEM method was used to visualize the dual-species biofilms and compare them with the *C. auris* biofilms after 24 and 48 h of culture (Figure 3a,b). The microscopic analysis of the *C. auris* biofilms showed a lack of hyphae and a weak ability to aggregate cells. Similarly, in comparison with the reference mono-species biofilm, the mixed-species biofilms of *C. tropicalis* and *C. glabrata* showed low hyphal abundance and weak aggregation. In contrast, the *C. auris*/*C. krusei* biofilms were characterized by a more abundant growth of predominantly planktonic cells with more intensive cell aggregation. Only in the case of the combination of *C. auris* and *C. albicans* was very intensive growth of hyphae-rich biofilms observed. After 48 h of culture, a loss of biofilm biomass was visible in each analyzed probe (Figure 3b).

**Figure 4:**
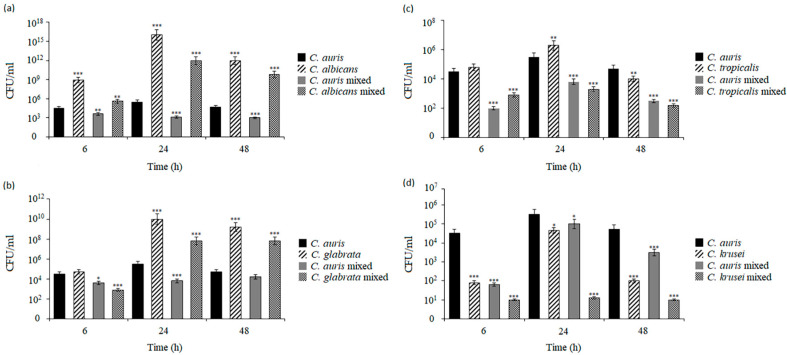
Total cell numbers (CFU/mL) from mono-species and dual-species biofilms of *C. auris* and other *Candida* strains: (**a**) *C. auris* with *C. albicans*, (**b**) *C. auris* with *C. glabrata*, (**c**) *C. auris* with *C. tropicalis*, and (**d**) *C. auris* with *C. krusei*. The analyses were performed at 6, 24, and 48 h at 37 °C. At each time point of biofilm maturation, the CFU/mL of *Candida* from the mono-species and dual-species biofilms were compared with the CFU/mL of *C. auris* from the mono-species culture (black columns, * *p* < 0.05; ** *p* < 0.01; *** *p* < 0.001) by Student’s *t*-test. Data are the means ± SD of experiments performed in of three duplicates and repeated two times.

The microscopic observations of biofilms confirmed visual differences in the structure of the biofilm organism but were not sufficient to determine the quantitative relationships between the two species forming these structures. Therefore, the colony-forming units (CFUs) of each *Candida* species in the mono-species or dual-species biofilms were determined. The colonies of each species that grew on chromogenic media were counted after 6 h (early biofilms), 24 h (intermediate biofilm), and 48 h (mature biofilms). Figure 4 shows the mean CFU/mL values obtained for the mono-species and dual-species biofilms of *C. auris* and other *Candida* species. Compared to the CFU/mL of *C. auris*, the differences were observed after 6 h of incubation in both single-species and dual-species *Candida* biofilms. There were no such differences in the mono-species *C. glabrata* and *C. tropicalis* biofilms. At 24 h, the CFU/mL values of *C. albicans*, *C. glabrata*, and *C. krusei* were significantly different from that in the *C. auris* reference strain (*** *p* < 0.001), while this difference in the case of *C. tropicalis* was smaller (** *p* < 0.01). In the mixed *C. auris*/*C. albicans* and *C. auris*/*C. glabrata* biofilms, species other than *C. auris* dominated, whereas *C. auris* was the dominant species in the mixed *C. auris*/*C. tropicalis* and *C. auris*/*C. krusei* biofilms although the initial concentrations of the two species in the inocula were the same. After 48 h of incubation, all the non-*Candida auris* biofilms were significantly different from the *C. auris* biofilms. In the *C. auris*/*C. albicans* and *C. auris*/*C. glabrata* biofilms, *C. albicans* and *C. glabrata* cells predominated (*** *p* < 0.001). In contrast, the *C. auris*/*C. tropicalis* and *C. auris*/*C. krusei* biofilms were dominated by *C. auris* cells (*** *p* < 0.001). In all the co-cultured biofilms, the total CFU/mL value of each organism was significantly different from the total CFU/mL value of their corresponding mono-species *Candida* biofilms within 6–48 h of biofilm formation. The cell numbers from the biofilms confirmed the visual differences in the biofilm constitution.

### 3.4. Candida Species Interactions in the In Vivo Model

**Figure 5:**
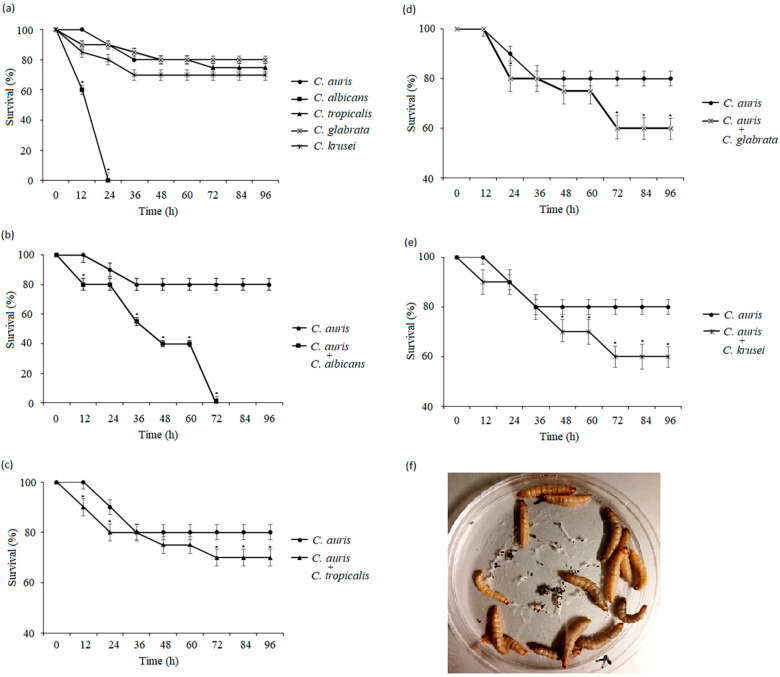
Survival curve of *G. mellonella* larvae infected with *Candida* strains: (**a**) larvae infected with single infections; (**b**) larvae infected with *C. auris* (single infection) and compared with larvae infected with *C. auris* and *C. albicans;* (**c**) *C. auris* and *C. tropicalis*; (**d**) *C. auris* and *C. glabrata*; and (**e**) *C. auris* and *C. krusei* (mixed infections). (**f**) Representative photo of *G. mellonella* larvae from a Petri dish culture.

To verify the interspecific interactions between *C. auris* and the other *Candida* species in vivo, we tested the pathogenicity of each *Candida* species alone and in combination with the *G. mellonella* model. The single infections with *C. auris*, *C. albicans*, *C. tropicalis C. glabrata*, or *C. krusei* after inoculation of *G. mellonella* larvae with cells at a concentration of 2 × 10^6^ CFU/larva showed 100% larval mortality following a period of 24 h post-infection as a result of *C. albicans* infection. Larvae infected with *C. auris* exhibited 20% mortality at the end of the experiment. In turn, the other species *C. tropicalis*, *C. glabrata*, and C. *krusei* were also less pathogenic to the *G. mellonella* larvae, with a mortality rate of 25%, 20%, and 30%, respectively, at the end of the experiment (Figure 5a). In order to study the interaction of *Candida* species in mixed infections in the *G. mellonella* model, larvae were infected with 1 × 10^6^ CFU/larva of *C. auris* and 1 × 10^6^ CFU/larva of *C. tropicalis*, *C. glabrata*, or C. *krusei*. A significant decrease in the *G. mellonella* survival rate was observed among larvae infected with heterotypic suspensions, compared with the groups infected with *C. auris* monotypic suspensions. The mixed infection with *C. auris* and *C. albicans* caused 100% mortality after 72 h post infection (Figure 5b). When the larvae were inoculated with *C. auris* and *C. tropicalis*, *C. auris* and *C. glabrata*, and *C. auris* and *C. krusei*, their viability decreased to 70%, 60%, and 60%, respectively, at the end of the experiment (Figure 5c,e). Figure 5f shows an example photo of the *G. mellonela* larval culture on a Petri dish.

These experiments were repeated at least twice and representative experiments are presented. At each time point, the viability of larvae infected with *Candida* mono-species was compared with the reference infection with *C. auris*, and the viability of larvae infected with dual-species infections was compared with the reference infection with *C. auris* (* *p* < 0.05) using the Student’s *t*-test. Data are means ± SD of experiments repeated three times.

**Figure 6:**
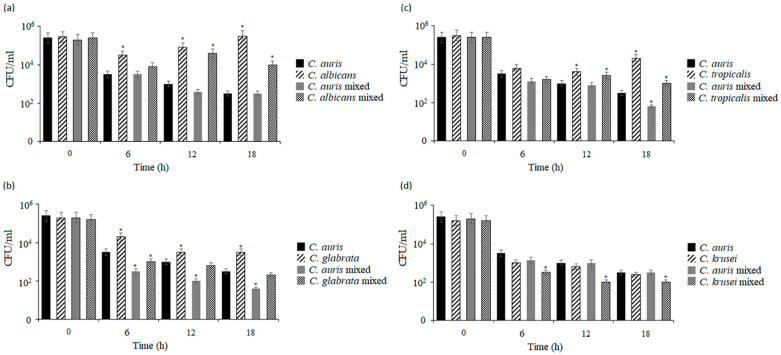
Total cell numbers (CFU/mL) of *Candida* species in *G. mellonella* hemolymph at various time points of dual-species experimental infections: (**a**) *C. auris* with *C. albicans*, (**b**) *C. auris* with *C. glabrata*, (**c**) *C. auris* with *C. tropicalis*, and (**d**) *C. auris* with *C. krusei.* At each time point of infection, the CFU/mL of *Candida* from mono-species and dual-species infections were compared with the CFU/mL of *C. auris* from mono-species infections (* *p* < 0.05) using the Student’s *t*-test. Data are the means ± SD of experiments performed in three repeats.

In turn, the measurements of *Candida* CFU/mL in the *G. mellonella* hemolymph showed different growth patterns in the *C. auris* and *C. albicans* co-infection variants in both single and mixed infections. A statistically significant difference was found between the *C. auris* single infection groups and the single and double infections with *C. albicans* at 6, 12, and 18 h after fungal inoculation, where *C. auris* showed noticeably weaker growth both in comparison to the monotypic *C. albicans* infection and in the *C. auris-C. albicans* interaction group compared to the monotypic group (* *p* < 0.05) (Figure 6a). In the mixed *C. auris-C. glabrata* and *C. auris-C. tropicalis* infections, the number of CFU/mL was also counted, confirming that *C. auris* showed significantly smaller increases in the interaction group, compared to the monotypic group at 6, 12, and 18 h in the mixed infection with *C. glabrata* (Figure 6b). Similar growth patterns were detected in the mixed infections with *C. tropicalis* at 6 and 12 h, although a statistically significant difference was observed between the groups at 18 h post-infection (* *p* < 0.05) (Figure 6c). In contrast, in the *C. auris-C. krusei* co-infection, there were no statistically significant differences between the monotypic *C. auris* infection and the heterotypic infection. Noticeable differences were found in the number of CFU/mL of *C. auris* in the monotypic infection and the number of CFU/mL of *C. krusei* in the mixed infections at 6, 12, and 18 h after fungal inoculation (* *p* < 0.05) (Figure 6d). Based on these results, the analyzed survival curves, and the obtained CFU/mL values, it can be confirmed that the *G. mellonella* groups with mixed infections showed a higher infection rate, compared to the groups with single *C. auris* infections, which indicates cooperation between *Candida* species.

## 4. Discussion

Although *C. albicans* remains the leading *Candida* species responsible for infections, the clinical significance of species referred to as non-albicans *Candida* is also growing. Recently, the etiology of infections caused by *Candida* species changed when a new species, *C. auris*, was first described in the medical literature [45]. Its clinical significance is systematically increasing, as evidenced by the recognition of *C. auris* as one of the greatest threats to public health. In addition to the rapid spread of *C. auris* strains worldwide, an important aspect of the threat associated with this species is its high epidemic potential and the ability to survive on abiotic surfaces and contaminate the hospital environment. Unlike other pathogenic yeast, *C. auris* exhibits a high potential for environmental transmission and horizontal transmission (between patients) with possible long-term colonization of the human body (>one year) and with the potential to cause invasive infections [46,47]. *C. auris* has a number of adaptations to environmental niches and to the host organism, including morphological variability, production of lytic enzymes, adaptive mechanisms in stressful environmental conditions, biofilm formation, and resistance to antifungal drugs [48,49,50].

The emergence and spread of a new pathogen in the environment is associated with the creation of new relationships and interactions between other *Candida* species present in the same niches. In this study, we evaluated the interactions between *C. auris* and four common *Candida* species in both in vitro and in vivo models.

The in vitro interactions between different *Candida* species were evaluated in biofilms formed at the bottom of 96-well plates. The growth patterns of the monotypic biofilms of individual *Candida* species varied because the biofilm-forming ability and structure are microbial strain- and species-dependent [51]. Of all the medically important *Candida* species, *C. albicans* is the largest and most important biofilm producer. Biofilms formed by *C. albicans* have a more heterogeneous structure, consisting of yeast cells, hyphae, and pseudohyphae surrounded by an extracellular matrix (ECM) [52]. Therefore, in our study, the metabolic activities of *C. albicans* biofilms differed significantly from other species and were significantly higher. Compared to the *C. albicans* biofilms (after 6 h of the experiment) and the *C. glabrata* biofilms (after 18 h of the experiment), the *C. auris* biofilms showed significantly lower metabolic activity during biofilm growth. However, compared to the *C. krusei* biofilms, this activity was significantly higher during 48 h of the experiment. When comparing the mono-species *C. auris* biofilms with the *C. tropicalis* biofilms, *C. tropicalis* metabolic activity predominated after 18 h, and *C. auris* metabolic activity predominated after 36 h of biofilm formation. Previously, Sherry et al. (2017) have shown that *C. auris* has the ability to form biofilm in vitro although its biomass was weaker than that of *C. albicans* biofilm. Furthermore, the ability to form biofilm is dependent on the strain and the morphological/phenotypic type. *C. auris* was initially thought to be incapable of growing as true hyphae or pseudohyphae, which are essential for the invasion of host tissues [53]. Yue et al. (2018) demonstrated the existence of three types of cells produced by *C. auris*: typical yeast cells, filamentation-competent yeast cells, and a filamentous form [54]. In our study, the SEM micrographs showed that *C. auris* grew in biofilms as aggregated yeasts without hyphae. Borman et al. (2016) observed the occurrence of aggregating and nonaggregating forms of *C. auris* in vitro and studied the pathogenicity of this yeast using the *G. mellonella* model. The aggregating forms were characterized by lower virulence, while the nonaggregating forms were as virulent as *C. albicans* [55]. Similar results associated with higher virulence of nonaggregating forms were obtained by other researchers [53,56,57]. In addition, Garcia et al. (2021) found the ability of *C. auris* to produce filaments in an in vivo infection model, but this property was not necessarily the main determinant of *C. auris* pathogenicity, considering that the less virulent aggregating forms show a stronger ability to form pseudohyphae. The lower virulence of aggregating forms may be associated with their weaker ability to spread in the host organism [57]. Wurster et al. (2019) published different results, as they reported the lack of a correlation between the ability of *C. auris* to aggregate and virulence in the *Drosophila melanogaster* infection model. Eventually, the ability of aggregating and non-aggregating forms to form biofilm was found [58]. As shown in our study, the mixed biofilms of *C. auris* with *C. albicans* and *C. glabrata* exhibited higher metabolic activity than the *C. auris* mono-species biofilms. In turn, the mixed biofilms of *C. auris* with *C. tropicalis* showed higher metabolic activity only up to 18 h of biofilm growth, compared to the mono-species biofilms of the reference species. In contrast, the dual-species *C. auris-C. krusei* biofilm showed significantly lower activity for 48 h of growth, compared to the biofilm of *C. auris*. Furthermore, lower CFU/mL values of *C. auris* were found in the mixed biofilms with *C. albicans*, *C. tropicalis*, *C. glabrata*, or *C. krusei*, compared to the single biofilms formed only by *C. auris.* The reduction in CFU/mL of *C. auris* observed in the mixed biofilms in relation to the single biofilm suggests that *C. auris* establish competitive interactions with other *Candida* species during biofilm formation. On the other hand, the CFU/mL values of *C. auris* in the dual-species biofilms were lower than the CFU/mL of *C. albicans* or *C. glabrata*, indicating quantitative dominance of these species in the mixed biofilms. In turn, *C. tropicalis* and *C. krusei* appeared to be dominated by *C. auris* in the mature mixed biofilms. Previously, studies of interactions between *Candida* species in mixed biofilms were focused on *C. albicans* and their coexistence with other yeast species. In the present study, *C. albicans* dominated in the co-culture biofilms although the initial concentrations of both species in the inoculum were the same. These results are in contrast to those presented by El-Azizi et al. (2004), who reported that the addition of *C. albicans* to preformed biofilms of *C. krusei*, *Candida lipolytica*, or *Candida guilliermondii* and co-incubation of *C. albicans* with other *Candida* species, such as *C. krusei*, *C. glabrata*, *C. lipolytica*, *C. guilliermondii*, or *C. parapsilosis*, reduced the number of *C. albicans* cells in the resulting mixed biofilms [32]. Similarly, Kirkpatrick et al. (2000) also found competitive interactions between *C. albicans* and *C. dubliniensis* in mixed biofilms [37], while Silva et al. (2011) found neutral interactions between *C. albicans* and *C. glabrata* in biofilms formed on acrylic resin [51]. In turn, Pathak et al. (2012) and Thein et al. (2000) observed reduced growth of *C. albicans* during the interaction between *C. albicans* and *C. krusei* in biofilms formed in vitro [42,59]. As reported by Martins et al. (2016), *C. albicans* and *C. glabrata*, *C. parapsilosis*, *C. krusei*, *C. tropicalis*, and *C. rugosa* could co-exist in biofilms displaying apparent antagonism [41].

The present study is the first in vivo study that attempts to investigate the interactions between *C. auris* and different *Candida* species in the development of experimental candidiasis using the *G. mellonella* model. For the study in this model, the pathogenicity of each *Candida* species was tested initially in the *G. mellonella* larvae. Overall, 100% of larvae infected with *C. albicans* monotypic suspensions died after a period of 24 h post-infection. *C. auris***,** *C. glabrata***,** *C. tropicalis*, and *C. krusei* were less pathogenic, with mortality rates ranging from 20% to 30%, respectively, 96 h after inoculation. These findings are consistent with those reported by Wang et al. (2018). The authors demonstrated significantly lower virulence of *C. auris* than that of *C. albicans* using the *G. mellonella* model [60]. In a study conducted by Fan et al. (2021) with *G. mellonella* larvae, greater virulence of filamentous and elongated forms of *C. auris* was demonstrated [61]. Junqueira et al. (2011) found *G. mellonella* mortality rates of 100% for *C. albicans*, 25% for *C. krusei*, and 20% for *C. glabrata* [62]. Cotter et al. (2003) confirmed that *C. albicans* was the most pathogenic species, causing the death of 90% of larvae, while the mortality rate for the other species was 70% for *C. tropicalis*, 45% for *C. parapsilosis*, 20% for *C. krusei*, and 0% for *C. glabrata.* These data confirm that non-albicans species, including *C. auris*, exhibit lower pathogenicity in the *G. mellonella* model than *C. albicans* species [63]. In the groups with mixed *Candida* infections, a decreased *G. mellonella* survival rate was observed, in contrast to the groups infected with monospecific *C. auris*. The *C. auris* single infection caused 80% mortality at 36 h post-infection. On the other hand, 100% mortality rates were reached at 24 h after the mixed infections with *C. auris* and *C. albicans*, while 70%, 60%, and 60% were reached at 96 h after the mixed infections of *C. auris* with *C. tropicalis*, *C. auris* with *C. glabrata*, and *C. auris* with *C. krusei*, respectively. These results suggest that, during infections in *G. mellonella*, there was a non-competitive interaction between *C. auris* and the other *Candida* species tested, which is identical to the results obtained in the *C. auris-C. albicans*, *C. auris*-*C. glabrata*, and *C. auris*-*C. tropicalis* (up to 24 h of infection) biofilms formed in vitro and opposite to the results obtained in the *C. auris*-*C. krusei* and *C. auris*-*C. tropicalis* (after 24 h of infection) biofilm cultures in vitro in the present study.

The quantification of *Candida* species CFU/mL in *G. mellonella* hemolymph in single infections evidenced that the amount of yeast cells recovered immediately after the inoculation (time 0) was not different from the amount used to initiate the infection. However, a reduction in the number of recovered cells was found 6 h following the inoculation. This effect was probably due to the host immune response. After this period, the larval immune system was not able to fight only the *C. albicans* infection; thus, the CFU/mL value gradually increased, reaching a peak at 18 h post-infection, which could justify the 100% mortality rate found in the 24 h period. The total CFU/mL value of *C. auris* and other non-*albicans* species measured in the larval hemolymph indicated that the immune system of this host was able to reduce and maintain the fungal load after 6–18 h infection, which may explain the lower mortality of these species compared to *C. albicans* observed in the survival tests. Regarding the CFU values in the hemolymph after the mixed infections, we showed that the *C. auris* yeast levels were significantly higher in the single infections than those found in the mixed infections with *C. glabrata* within 18 h of testing at each time point and the mixed infections with *C. tropicalis* after a period of 18 h post-infection. In turn, during the mixed infections using *C. auris* with *C. albicans* and *C. auris* with *C. krusei*, the *C. auris* CFU levels were similar to the CFU values in the single *C. auris* infections, suggesting that the presence of these non-*C. auris* species in *G. mellonella* results in more lethal infections. Previously, *G. mellonella* larvae were used by Rossoni et al. (2015) to evaluate the interactions between *C. albicans*, *C. krusei*, and *C. glabrata* in mixed infections. The authors analyzed the survival rate and quantification of fungal cells in the hemolymph and determined the competitive interactions of *C. albicans* with *C. krusei* and *C. glabrata* during biofilm formation and the development of experimental candidiasis [40].

The present study is a piece of pioneering research on the development of mixed infections of *C. auris* with other common pathogenic *Candida* species. According to our findings, *C. auris* interacts both competitively or noncompetitively with other species, depending on the coexistent *Candida* species, the phase of the biofilm formation in vitro, or the stage of infection in vivo. These studies confirmed that *C. auris* biofilms are capable of persisting in the environment with other *Candida* species, and it is likely that these infections will be heterogeneous with other *Candida* species in organisms, in the environment, and on medical devices. Recognition of the heterogeneity of yeast infections is primarily associated with biofilm formation and may influence treatment decisions, especially in patients who do not respond to therapy.

In our opinion, further studies are also necessary to investigate the interactions between *Candida* species using a larger number of *Candida* strains. This study was performed only with laboratory reference strains and these results should be extended to clinical isolates, as *C. auris* clinical isolates are known to show intraspecific variability in relation to pathogenicity in vitro and in animal models.

In conclusion, we have shown in this study that *C. auris* is able to survive in the environment and coexist with other clinically important *Candida* species that are responsible for fungal biofilm-associated infections in hospital settings. Such information sheds light on the interactions of *Candida* species in the biofilm community and in co-infection and may lead to new approaches for the control of such diseases.

## Data Availability

Data are contained within the article.
